# When the Well is Dry, We Know the Worth of Water

**DOI:** 10.3389/fped.2016.00012

**Published:** 2016-02-25

**Authors:** David Epstein

**Affiliations:** ^1^Department of Anesthesiology Critical Care Medicine, Children’s Hospital Los Angeles, Keck School of Medicine of the University of Southern California, Los Angeles, CA, USA

**Keywords:** work–life balance, self-reflection, self-perception, expectation, well-being

When I was asked to write an article about work–life balance, I told my wife and she innocently asked me, “In order to write about work–life balance, don’t you need to have a work–life balance first?” After all of these years of practice, didn’t I have that balance worked out? Was my perception totally inaccurate? I know that struggling to balance my work and life outside of work has been something that I have been keenly aware of for some time. Nevertheless, it seems as though I have more work to do! And gathering from the request to write a piece on this topic, I am not alone in this struggle. So, I started contemplating the etiology of the work–life balance problems, when I noticed the importance of work–life balance, the benefits of and obstacles to work–life balance, and how I would try to solve the problem for myself.

The struggle between competing priorities is the crux of the work–life balance problem. But, in order to have a struggle, one must realize that there is a problem and possess some degree of self-awareness. If there is no perceived problem, then a struggle will not exist and one would assume balance. Most of this perceived problem or lack of a problem is determined by one’s priorities. So, work–life balance appears to be a relative matter. The struggle originates from elements within one’s life that can be classified as biological, psychological, spiritual, and social. The reflective understanding, prioritization, and integration of these areas will determine whether or not a work–life balance can be achieved. This construct has made it a little easier for me to reflect, assess my own work–life balance, and welcome the idea that a work–life balance has great appeal but is almost impossible based on today’s work context and expectations.

Before the struggle to achieve work–life balance begins, there needs to be a life transition to recognizing the importance of work–life balance. Many of us grew up with an idealized notion of what it meant to be a physician. Our role models (whether they were family, friends, or other professionals) and other environmental exposures that may have been related to the media and entertainment have shaped our idealized notion of what it means to be a physician. We are also shaped by the society’s expectation of what it means to be a physician and what we must do. There is overlap in the context of theses exposures, but, in the end, it creates a unified picture or template in our mind about how we should be as a physician. I enjoyed watching television shows, such as *St. Elsewhere*, *ER*, and *House*. I witnessed the lives and interactions of real physicians in medicine, related to me or not. I saw evidence of the society’s expectations of physicians by scrutiny in the news and legal cases. While everyone generates their own, personalized picture of what a physician is, the image that was created in my mind was one of an individual who was extremely compassionate, dedicated to their work, shouldered a tremendous responsibility to heal people, and made *no* mistakes. For me, these attributes were only reinforced by my medical training with hours of studying, pressure to do well academically, pressure to perform well clinically, and the expected dedication to the field in the form of clinical, research, teaching, and administrative responsibilities. The rigors of pediatric residency and a critical care fellowship did little to change my course in development and only encouraged the idea of work, work, and work. Nevertheless, training in pediatrics did lend itself to a softer and more tempered training environment than I witnessed in other areas of medicine. Dealing with children in medicine, in my perception, tends to attract a certain type of person, because it is difficult to work with children if you are not patient and able to act like a kid yourself sometimes. Regardless of the softer and more tempered training environment, the pressure to be an amazing physician is amplified by a child’s life riding on the line and a parent’s expectation to protect their child from harm. This varies with the acuity of illness, but I believe that it is a more prevalent dynamic in the field of pediatric critical care. The foundation of these feelings can be traced to not only how you perceive yourself and how others perceive you, but also how you perceive your role and how others perceive your role. Understanding this takes a great deal of self-reflection, self-awareness, and introspection that comes with maturity, experience, and letting go of one’s ego. This paper reflects my own self-reflection and introspection. I believe that people reach the ability to contemplate these issues at different points in their lives and I can say with certainty that I could not have written this paper 10 years ago. As I began to appreciate the biological, psychological, spiritual, and social elements of my life, I became more aware of their importance and that forced me to prioritize them in relation to being a physician. Once that happened, my struggle began.

As a medical community, we know from studies that one’s overall health and well-being is affected by one’s physical health, mental health, belief system, and how one interacts with other human beings or integrates into society. These are avenues to either the benefit of one’s well-being or its deterioration. The Dalai Lama said, “In dealing with those who are undergoing great suffering, if you feel “burnout” setting in, it is best, for the sake of everyone, to withdraw and restore yourself. The point is to have a long-term perspective.” Physicians are not immune to illness, social isolation, depression, and other effects of lack of work–life balance. How often have we heard of physicians’ physical health being affected by the stress of work? How often have we heard of physicians getting divorced or committing suicide? The biological, psychological, spiritual, and social elements integrated into one’s life play an important role in one’s overall health (1–3). So, why should physicians in critical care be any different? The quote by Benjamin Franklin, “When the well’s dry, we know the worth of water,” seems especially apropos.

Despite us knowing the benefits of a balanced life, our society has created catalysts to unbalance our life. Part of the problem of work–life balance, in this day and age, is that technology has expanded ever so quickly that our lives are connected 24 h per day, 7 days per week. We live in a world that has created ways to do things faster, obtain information quicker, and stay connected continuously. There are very few people in developed countries who do not have a computer, handheld electronic device, smartphone or other method of communication, or way to perform work outside of the confines of their office, keeping them tethered to their work. This is not unique to medicine. The line between work and life is blurred. Work weaves into life and life weaves into work, such as checking work emails diligently on one’s smartphone at home and staying connected with friends outside of work through social media while one is at work (4, 5). We get distracted at work by our social media, phone calls, and personal emails and distracted at home by our work emails, project deadlines, and the ability to meet virtually from almost anywhere *via* FaceTime, Skype, Go-To-Meeting, or any other mode of visual communication. I have attended work meetings from home *via* FaceTime and I have seen my family from work *via* FaceTime. There is very little separation between one’s work and life these days. Some would go as far to say that there is no such thing as work–life balance in today’s world. Nevertheless, I have to believe that a work–life balance can exist because the prospect of having only to look forward to working makes for a very empty future.

In this day and age, I believe that identifying work–life balance is a personal journey, but work can possibly help as well. When I was growing up, my father worked hard and would have long work days, but when he was home … he was home. And, I did not think of him as the dentist that he was. He was just my father. As a father myself, I feel that my children know me as a physician and see me working at home … on the phone, checking emails, and working on the computer. I have tried to make more of a separation, but it is difficult. It goes back to how I perceive myself and my role, not only as a physician but also as a husband, father, son, etc., and how others perceive my roles and me. I feel the expectation to be at the top of my game clinically, be productive academically, and be an amazing teacher. While these things are measured at work, I place the importance on them. The importance of these work expectations conflict with life outside of work expectations because they both take time and investment of energy. One cannot pour time and energy into one thing without taking away from the other. Am I a physician first or am I a father/son/husband/friend/member of the community first? Do I take care of others or do I take care of myself? Do I believe in integrating various elements into my life or do I commit myself solely to medicine? Only I can answer these questions. But, in the end, if I do want to live in both worlds with some success in each, I do need to find a balance. How I strike that balance is up to me, and I can imagine that there is no universal way to accomplish this, so each person needs to find what works for him or her. Our work also provides an opportunity to help us balance. While employment laws dictate that employees take breaks during work, this is not always practical in the pediatric ICU. The ICU environment can be a vortex of non-stop activity and demands. So, a culture of putting an emphasis on “down time” must be encouraged.

There is a billion dollar industry focused on self-improvement, leadership, and work culture with books, videos, podcasts, seminars, and many other mediums to spur improvements in one’s life and work, not to mention the advice of family, friends, acquaintances, impartial observers, therapists, and complete strangers writing articles about work–life balance. One should start at the beginning. As I mentioned, it is one’s priorities that dictate what should be done. This is determined by what one values and what one’s mission in life and work is. That is to say, “Why do I do the things that I do?” After that, one should envision where he or she wants to be … in 5 years, 10 years, etc. This sounds so familiar because we all have to assess these questions about where we want to be in 5 or 10 years with our work life, but not as often in our life outside of work. If one wants to find balance, these 5- and 10-year plans should include your multidimensional life and not just the one-dimensional work life and work. Once you realize where you are and where you want to end up in life, filling in the details and strategies is really an individualized journey.

So, back to the question, “Is work–life balance possible?” Work–life balance takes a concerted effort and work, not only in one’s personal life but also in one’s work life. Furthermore, work–life balance requires looking at oneself, critically, while assessing one’s life priorities, shedding perceptions/expectations, and giving oneself permission to seek balance and live a balanced life. As we go through our journey of life, our priorities may shift or even change, and how we balance work and life may shift or even change as well (6). Ultimately, with the self-reflection and desire to make an effort to balance one’s life, I believe that work–life balance is achievable.

## Author Contributions

The author confirms being the sole contributor of this work and approved it for publication.

## Conflict of Interest Statement

The author declares that the research was conducted in the absence of any commercial or financial relationships that could be construed as a potential conflict of interest.
